# Integrative network analysis reveals different pathophysiological mechanisms of insulin resistance among Caucasians and African Americans

**DOI:** 10.1186/s12920-015-0078-0

**Published:** 2015-02-07

**Authors:** Swapan Kumar Das, Neeraj Kumar Sharma, Bin Zhang

**Affiliations:** Section on Endocrinology and Metabolism, Department of Internal Medicine, Wake Forest School of Medicine, Medical Center Boulevard, Winston-Salem, NC 27157 USA; Department of Genetics & Genomic Sciences, Icahn Institute of Genomics and Multiscale Biology, Icahn School of Medicine at Mount Sinai, 1470 Madison Avenue, New York, NY 10029 USA

**Keywords:** Insulin resistance, Obesity, Co-expression network, Adipose tissue, Caucasian, African American, Transcript, Integrative genomics, Ethnic disparity

## Abstract

**Background:**

African Americans (AA) have more pronounced insulin resistance and higher insulin secretion than European Americans (Caucasians or CA) when matched for age, gender, and body mass index (BMI). We hypothesize that physiological differences (including insulin sensitivity [S_I_]) between CAs and AAs can be explained by co-regulated gene networks in tissues involved in glucose homeostasis.

**Methods:**

We performed integrative gene network analyses of transcriptomic data in subcutaneous adipose tissue of 99 CA and 37 AA subjects metabolically characterized as non-diabetic, with a range of S_I_ and BMI values.

**Results:**

Transcripts negatively correlated with S_I_ in only the CA or AA subjects were enriched for inflammatory response genes and integrin-signaling genes, respectively. A sub-network (module) with *TYROBP* as a hub enriched for genes involved in inflammatory response (corrected p = 1.7E-26) was negatively correlated with S_I_ (r = −0.426, p = 4.95E-04) in CA subjects. S_I_ was positively correlated with transcript modules enriched for mitochondrial metabolism in both groups. Several S_I_-associated co-expressed modules were enriched for genes differentially expressed between groups. Two modules involved in immune response to viral infections and function of adherens junction, are significantly correlated with S_I_ only in CAs. Five modules involved in drug/intracellular transport and oxidoreductase activity, among other activities, are correlated with S_I_ only in AAs. Furthermore, we identified driver genes of these race-specific S_I_-associated modules.

**Conclusions:**

S_I_-associated transcriptional networks that were deranged predominantly in one ethnic group may explain the distinctive physiological features of glucose homeostasis among AA subjects.

**Electronic supplementary material:**

The online version of this article (doi:10.1186/s12920-015-0078-0) contains supplementary material, which is available to authorized users.

## Background

Ethnic differences exist in prevalence of common metabolic disorders, including type 2 diabetes (T2D) and metabolic syndrome [1]. In the United States, the recently estimated age-adjusted prevalence of T2D in adults is 7.6% among non-Hispanic Caucasians (European Americans or CA), and 14.9% among non-Hispanic African Americans (AA) [2]. Clinical (visceral adiposity and waist circumference) and biochemical (markers of inflammation) factors only partly explain these ethnic disparities [3-5]. The physiology of glucose homeostasis in African Americans is distinctive, with more pronounced insulin resistance, higher insulin secretion, and reduced hepatic insulin clearance compared to CAs [6]. These ethnic differences are significant even after accounting for age, gender, and obesity. Differences in evolutionary history have distinctly shaped the genetic (and epigenetic) architecture of AA and CA populations and may explain the ethnic differences in the stabilization points (canalization) of insulin sensitivity and other glucose homeostasis phenotypes. A recent study suggested that this ethnic stratification could be implicated in the different natural courses of T2D onset and may lead to differences in T2D prevalence [7]. Identifying molecular mechanisms that can explain the observed ethnic differences in insulin sensitivity will be helpful in developing suitable prevention, surveillance, and treatment strategies for the pathogenesis of T2D. However, the genetic cause or the physiological mechanisms for greater insulin resistance among AAs are unknown [8].

Transcript expression in human tissues is correlated with insulin sensitivity and linked to dysregulation of genes involved in the pathophysiology of obesity, insulin resistance, and T2D [9]. Lymphoblastoid cell line transcript profiles of different ethnic groups from the HapMap project also identified significant ethnic differences in expression of transcripts [10-13]. Comparisons of gene expression in subcutaneous adipose and skeletal muscle from a small cohort of CA and AA subjects by our laboratory and others indicated ethnic differences in transcript expression; some of these transcripts are associated with insulin sensitivity [14,15]. However, no analyses have identified transcriptional mechanisms associated with insulin sensitivity and related metabolic traits that predominate in one ethnic group compared to another.

Expression of transcripts involved in the same biological function tend to be co-regulated by similar factors (genetic or environmental) and can be identified as distinct network modules, where genes within a module are more highly interconnected (correlated) with each other than genes in other modules [16]. Thus, we hypothesize that distinctive physiological differences, including differences in insulin sensitivity (S_I_) between CAs and AAs, can be explained at least partially by gene expression network modules in tissues involved in glucose homeostasis. These modules may show characteristic patterns of insulin resistance-associated derangements predominantly in one ethnic group compared to others.

In this study, we systematically analyzed genome-wide expression data from subcutaneous adipose tissue of 99 CA and 37 AA non-diabetic subjects. A pressing task in analyzing omics data like those generated by this study is to identify and visualize a global landscape of interactomes that contribute to clinical endpoints such as disease onset and progression. Weighted gene coexpression network analysis (WGCNA) has emerged as a way to solve this problem through identification of gene modules comprised of highly interconnected genes over a gene-gene interaction heatmap [17]. WGCNA has been widely used to identify pathways and gene targets for a variety of common human diseases such as cancer [18,19], atherosclerosis [20,21], Alzheimer’s disease [16], obesity and diabetes [22,23]. A key contribution of this work is the integration of differential expression analysis, gene-trait correlation analysis, and gene network analysis to identify both the coexpressed gene modules that contribute to insulin sensitivity and the key causal regulators. Utilizing this integrative network approach, we identified transcript co-expression modules correlated with S_I_ and other related metabolic phenotypes in each ethnic group. Several modules enriched for important biological pathway genes showed differential connectivity between ethnic groups. Our study successfully delineated transcriptional networks associated with insulin sensitivity and other glucose homeostasis traits that are deranged predominantly in one ethnic group, and may explain differences in insulin sensitivity between CAs and AAs.

## Methods

### Ethics statement

All participants provided written informed consent under protocols originally approved (IRB #53028) by the University of Arkansas for Medical Sciences (UAMS). Our current study was approved by the Institutional Review Board at Wake Forest School of Medicine (IRB#00011083).

### Study subjects

We used subcutaneous adipose tissue samples from 136 non-diabetic individuals. These subjects were recruited previously; detailed methods for recruitment, physical examination, physiological experiments, and obtaining biopsies have been published [24]. In brief, Caucasian (European-American) or African-American men and women who were generally in good health, between 19 and 60 years of age, and had a BMI between 19 and 45 kg/m^2^ were recruited. All participants had a screening visit, at which time height, weight, and waist and hip circumference were measured; body fat was determined by dual x-ray absorptiometry (DXA) scan (Hologic QDR-4500); fasting blood samples for lipid measurements were taken; and a standard 75-g oral glucose tolerance test (OGTT) was done, with measurement of glucose and insulin at baseline and 30- minute intervals for 2 hours. OGTT data were analyzed by homeostatic model assessment [25]. Based on their OGTT results, individuals who did not have diabetes were selected for this study.

At the second visit, adipose biopsies were obtained from subjects under fasting conditions. Insulin modified (0.03 U/kg) frequently sampled intravenous glucose tolerance tests (FSIGT) were performed as described previously [14,24]. We used the MINMOD Millennium program to analyze FSIGT data to determine insulin sensitivity (S_I_) and acute insulin response (AIR_G_) [26]. Biopsies were obtained from abdominal subcutaneous fat near the umbilicus under local anesthesia (Lidocaine) using a Bergstrom needle. Each biopsy is made by opening the Bergstrom needle and applying suction using a 120 ml syringe. Samples were rinsed immediately in sterile normal saline, quick-frozen in liquid nitrogen, and stored at −80°C for further use. Demographic characteristics of our cohort are shown in Table 1.

**Table 1:** **Demographics of study population**

**Trait**	**Caucasian or European-American (CA)**	**African-American (AA)**
N	99	37
Gender (M/F)	42/57	21/16
Age (Yrs)	39.7 ± 10.9	42.1 ± 9.1
WHR	0.89 ± 0.19	0.89 ± 0.07
BMI (kg/m^2^)	28.2 ± 4.9	29.5 ± 6.3
% Fat mass	32.6 ± 9.0	29.6 ± 10.9
Triacylglycerol (mmol/l)	1.45 ± 1.36	1.14 ± 0.71
Total Cholesterol (mmol/l)	4.75 ± 0.92	4.47 ± 1.01
LDL Cholesterol (mmol/l)	2.75 ± 0.76	2.56 ± 0.89
HDL cholesterol (mmol/l)	1.39 ± 0.47	1.38 ± 0.33
Fasting Glucose (mmol/l)	4.74 ± 0.46	4.72 ± 0.53
2 h Glucose (mmol/L) ^¶^	5.80 ± 1.63	5.51 ± 1.91
Fasting Insulin (pmol/l)	43.6 ± 39.8	46.6 ± 61.2
HOMA-IR ^¶^	1.36 ± 1.34	1.50 ± 2.35
Matsuda Index ^¶^	7.60 ± 5.48	9.18 ± 6.12
S_I_ (×10^−4^.min^−1^ [μU/ml]^−1^)*	3.8 ± 2.3	3.4 ± 1.9
AIR_G_ (pmol/L)*	2860 ± 2177	4586 ± 3148
DI*	1515 ± 1144	2061 ± 1257

Continuous variables are shown as mean ± SD. ^¶^Metabolic measurements from OGTT; *Metabolic traits from MINMOD analysis of FSIGT evaluation of non-diabetic individuals. BMI, Body mass index; WHR, waist to hip ratio; LDL, Low density lipoprotein; HDL, High Density lipoprotein; HOMA-IR, homeostatic model assessment of insulin resistance; S_I_, insulin sensitivity index; AIR_G_, Acute insulin response to glucose; DI, Disposition index. Units are taken from MINMOD program.

### Laboratory measurements

Insulin was measured by the UAMS Clinical Research Center core laboratory using an immuno-chemiluminometric assay (Invitron Limited, Monmouth, UK). Plasma glucose was measured by glucose oxidase methods at LabCorp, Inc. (Burlington, NC). Plasma triglyceride, total cholesterol, and HDL cholesterol concentrations were measured directly (at LabCorp) by enzymatic colorimetric methods using an automated Hitachi Cobas C system and assay kits from Roche. LDL cholesterol and VLDL cholesterol concentrations were calculated indirectly by the Friedewald equation.

### RNA extraction

Total RNA was isolated from whole adipose tissue using the RNAeasy Lipid Tissue Mini kit (QIAGEN Inc-USA, Valencia, CA). The quantity and quality of the isolated total RNA samples were determined by ultraviolet spectrophotometry (Nanodrop, Thermo Scientific, Pittsburgh, PA) and electrophoresis (Experion nucleic acid analyzer, BioRad Laboratories, Inc., Hercules, CA), respectively. High-quality RNA with RIN (RNA integrity number) >8 was used for genome-wide transcriptome analysis.

### Microarray studies

Genome-wide transcriptome analysis and initial array processing were done at the Center for Human Genomics Core Laboratory (Wake Forest School of Medicine) using HumanHT-12 v4 Expression BeadChip (Illumina, San Diego, CA) whole genome gene expression arrays according to the vendor-recommended standard protocol [24]. In brief, biotin-labeled cRNA was prepared from 500 ng of total RNA using the Illumina TotalPrep RNA amplification kit, and 750 ng of biotinylated cRNA was hybridized to the chip in a hybridization oven at 58°C for 18 hours. Chips were processed through a series of posthybridization washes, blocked in blocking buffer, and moved to streptavidin-Cy3-containing detection buffer. Chips were then washed, dried, and scanned in the Illumina BeadArray Reader. All RNA samples were processed using the same lot of chips and reagents, and all experiments were performed within a month to avoid technical variability. Raw expression intensity was background subtracted and normalized by the average normalization algorithm as implemented in GenomeStudio Gene Expression Module v1.0 application software (Illumina). Normalized data were used for further analysis. Data for 17,434 probes annotated as an Entrez gene and with a detection p-value of ≤0.01 in 90% of the samples were used for final analysis. All expression data has been deposited in Gene Expression Omnibus (GEO; http://www.ncbi.nlm.nih.gov/projects/geo/) under accession number GSE65221.

### Real-time qPCR validation

Total RNA (1 μg) was reverse transcribed using a Qiagen QuantiTect reverse transcription kit (QIAGEN Inc., Valencia, CA) according to the manufacturer’s protocol. Transcript-specific oligonucleotide primers for quantitative real-time PCR (qRT-PCR) were designed to capture splice variants captured by the probe sequences in the array [14,24]. Selected transcripts were measured by qRT-PCR using Power SYBR green chemistry (Applied Biosystems, Inc., Foster City, CA) and normalized to the expression of human ribosomal protein, large P0 (RPLP0 or 36B4) gene [24]. The standard curves were generated for absolute quantification using pooled cDNA from the samples assayed.

### Statistical and bioinformatic data analysis

To identify differential expression of individual transcripts between CA and AA subjects, normalized gene-expression data from adipose tissue were first adjusted using linear regressions for age and gender (expression ~ age + gender). The residuals following regression were analyzed by Student’s *T*-test to identify transcripts with differential expression between CA and AA samples. Differentially expressed transcripts were defined as those with a ≥1.2 fold change and a p value ≤ 0.05. The residual expression values after covariate adjustments (for age and gender) were used for further testing, including network construction and gene-expression physiological phenotypic correlations. We used Spearman’s correlation coefficient to identify correlation of each transcript with S_I_. False discovery rates (FDR) were also calculated; a transcript was considered associated with S_I_ if FDR ≤0.05.

The CA and AA data sets from all 17,434 expressed probes were independently processed through the weighted gene co-expression network analysis (WGCNA) [17,27]. The weighted network analysis began with a matrix of the Pearson correlations between all gene pairs, and then converted the correlation matrix into an unsigned adjacency matrix using a power function, so that the resulting adjacency matrix, i.e., the weighted coexpression network, is approximately scale-free. To explore the modular structures of the co-expression network, the adjacency matrix was further transformed into a topological overlap matrix (TOM) [17]. Because topological overlap between two genes reflects both their direct interaction and their indirect interactions through all other genes in the network, this approach helps create more cohesive and biologically more meaningful modules. To identify modules of highly coregulated genes, we used average linkage hierarchical clustering to group genes based on the topological overlap of their connectivity, followed by a dynamic cut-tree algorithm to dynamically cut clustering dendrogram branches into gene modules. To distinguish between modules, each module was assigned a unique color identifier. The gene with the highest intramodular connectivity was considered the “hub gene” (most connected gene).

To examine how each gene module was related to gluco-metabolic traits (e.g., S_I_) we first performed principal component analysis for each module, and then computed module-trait correlation between the first principal component (module eigengene) and each trait [16,28]. The significance (p value) and false discovery rate (FDR) of each correlation were also calculated. The FDR was estimated through random permutation of sample names of the trait data. A module was associated with a trait if the FDR was ≤0.05. Overrepresentation of canonical pathways and biological processes in modules was measured through Fisher’s exact test and corrected for numbers of modules and functional categories tested. Ingenuity Pathway Analysis (IPA) also was used to reveal enrichment of canonical pathways within selected modules.

To quantify differences in adipose tissue transcript network organization between CA and AA subjects, we employed a modular differential connectivity (MDC) metric [16]. In brief, MDC is the ratios of the connectivity of all gene pairs in a module from CA subjects, to that of the same gene pairs from AA subjects. MDC is a continuous measure ranging from 0 to infinity. MDC > 1 indicates gain of connectivity or enhanced co-regulation between genes, whereas MDC < 1 indicates loss of connectivity or reduced co-regulation between genes. The statistical significance of the MDC metrics was computed through the FDR procedure. The significance or FDR of the MDC statistic can be accessed by permuting the data underlying the two networks. We estimated FDR based both on shuffled samples (i.e., networks with nonrandom nodes but random connections) and shuffled gene labels (i.e., networks with random nodes but nonrandom connections), and then selected the larger value as the final FDR estimate.

To further identify key regulators (drivers) genes of the modules identified by WGCNA, we applied the key driver analysis [16] to the module-based unweighted coexpression networks derived from ARACNE (Algorithm for the Reconstruction of Accurate Cellular Networks) [29]. ARACNE was used first to identify significant interactions between genes in each module based on their mutual information, and then remove indirect interactions through data processing inequality (DPI). For each ARACNE-derived unweighted network, we further identified the key regulators by examining the number of N-hob neighborhood nodes (NHNN) for each gene. For a given network, let μ be the numbers of NHNNs and d be the out degrees for all the genes. Genes with a number of NHNNs greater than $\overline{\mu}+\sigma \left(\mu \right)$ are nominated as key regulators [16]. This criterion identified genes with number of NHNNs significantly above the corresponding average value.

We also tested conservation of S_I_-associated modules between ethnic groups by analyzing overlap of module members and enrichment of modules for common or ethnic-specific S_I_-associated genes. Fisher’s exact test was used to quantitate significant overlap and enrichment. Modules in CAs that show significant overlap (FET_P ≤ 3.1E-06, i.e., Bonferroni corrected p value of 0.05) with a module in AAs and are enriched for S_I_-associated genes in both ethnicities were considered conserved, while modules enriched for genes associated with S_I_ in only one ethnic group were considered ethnic-specific.

We used two different methods to control for multiple testing issues: (1) the highly conservative Bonferroni correction method, to constrain the study-wise significance level; and (2) an empirical false discovery rate (FDR) method, which constrains the overall rate of false positive events. For instance, for the enrichment of functional categories in modules, we corrected for the number of modules and Gene Ontology processes and other categories tested. For the enrichment of expression clinical trait correlation signatures in modules, we used a combination of FDR <0.05 and nominal P < 0.05.

## Results

### Adipose tissue transcripts show ethnic differences in expression

**Figure 1:**
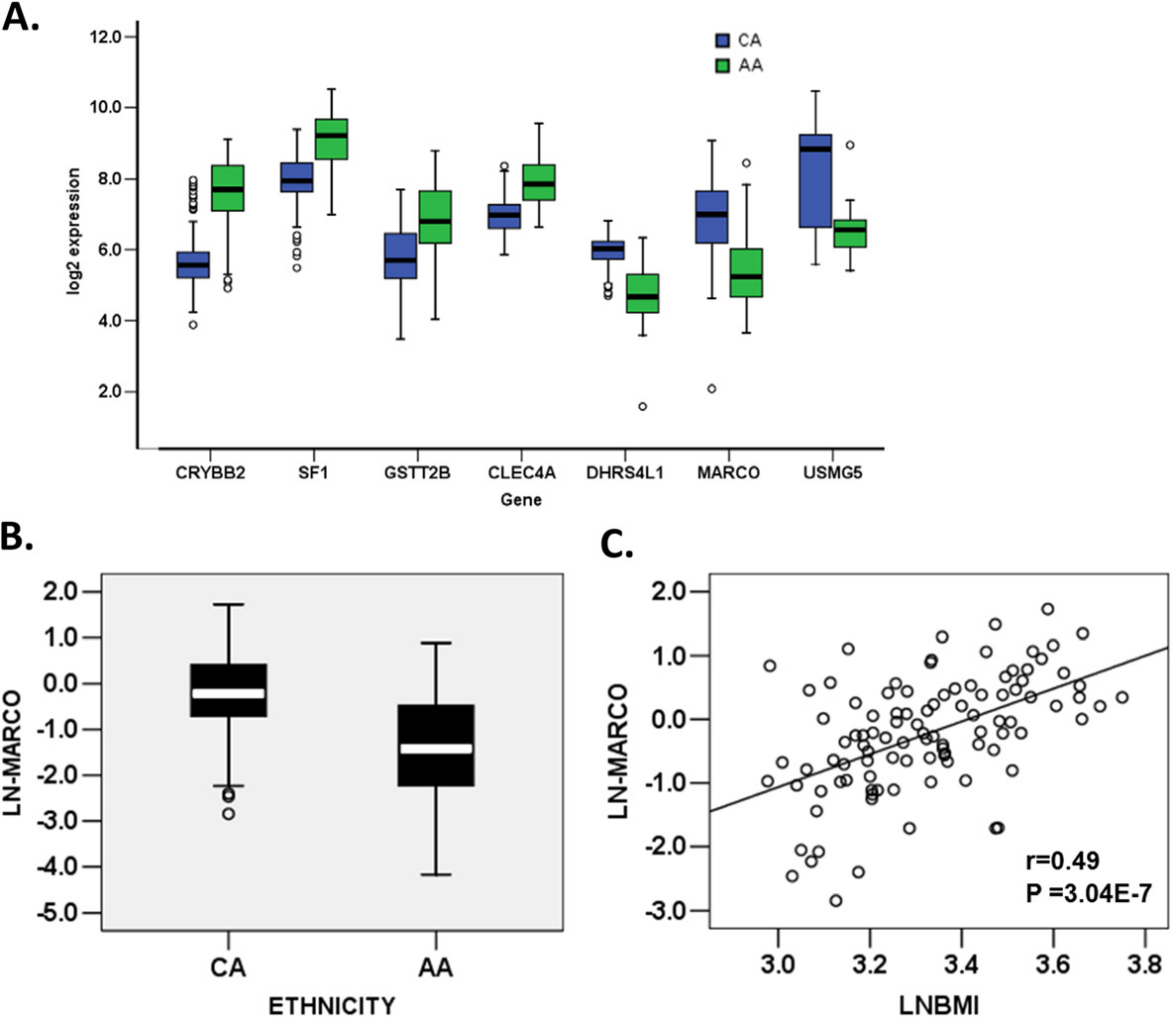
**Genes showing strong differential expression in adipose tissue of African Americans (AA) compared to Caucasians (CA). A)** Genome-wide expression microarray identified 2-fold higher expression of the *CRYBB2*, *SF1*, *GSTT2B*, and *CLEA4A* gene in AA subjects, while expression of *DHRS4L1*, *MARCO* (Macrophage receptor with collagenous structure), and *USMG5* was lower; **B)** Technical validation by qRT-PCR confirmed lower expression of *MARCO* gene in adipose tissue of AAs compared to CAs. *MARCO* expression is shown after 36B4 normalization and ln transformation. In A and B the box represents the interquartile range, which contains 50% of the values. The whiskers are lines that extend the box to the highest and lowest values, excluding outliers. A line across the box indicates the median; and **C)** *MARCO* expression is positively correlated with BMI in 99 non-diabetic CA subjects.

We identified significant differential expression (fold change 1.2 and p < 0.05) of 655 transcript probes between CA and AA subjects. These differentially expressed probes included 225 up-regulated and 193 down-regulated mRNA transcripts (Additional file 1: Table S1) of RefSeq genes with known function. Genes with >2-fold higher expression in AA subjects included crystallin-beta 2 (*CRYBB2*), splicing factor 1 (*SF1*), and C-type lectin domain family 4, member A (*CLEC4A*). Genes with >2 fold lower expression included diabetes-associated protein in insulin-sensitive tissue (DAPIT/*USMG5*), macrophage receptor with collagenous structure (*MARCO*), and dehydrogenase/reductase member 4 like 1 (*DHRS4L1*) (Figure 1A). We also validated differential expression of *MARCO* by qRT-PCR (Figure 1B) and detected a positive correlation of this gene with BMI in CA subjects (Figure 1C). Annotation of these differentially expressed genes using DAVID [30] indicated overrepresentation of these genes in important functional categories (Additional file 1: Table S2). Highly represented categories included genes involved in signal transduction and cell communication, endoplasmic reticulum, regulation of blood pressure, and the carboxylic acid biosynthetic process. In IPA analyses, enrichment of genes involved in glutathione-mediated detoxification and NRF2-mediated oxidative stress response pathways were among those differentially expressed in AA subjects.

**Figure 2:**
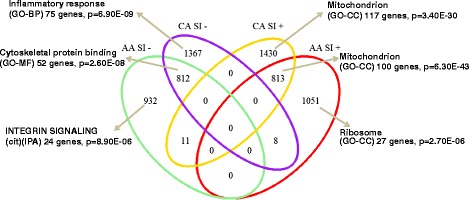
**Insulin sensitivity (S**_**I**_ **)-associated genes common and distinct for ethnic groups are enriched for important functional category and biological pathways.** Venn diagram showing overlap between Caucasian and African-American subjects for genes correlated (FDR ≤ 0.05) with S_I_ in subcutaneous adipose tissue. See Additional file 1: Table S3 for further details.

We identified significant correlations of 1,625 transcripts with insulin sensitivity (S_I_) in both CA and AA subjects (812 and 813 transcripts showed positive and negative correlations, respectively with S_I_). Some transcripts showed significant correlation (FDR ≤ 0.05) with S_I_ only in CA (n = 2,797 transcripts) or only in AA subjects (n = 1,983 transcripts) (Figure 2). These transcripts were enriched for important gene ontology categories and pathways (Additional file 1: Table S3 and Figure 2). Transcripts negatively correlated with S_I_ only in CA subjects (n = 1,367 transcripts) were enriched for inflammatory response pathway genes (75 genes, corrected p-value = 6.90E-09). Transcripts negatively correlated with S_I_ only in AA subjects (n = 932 transcripts) were enriched for integrin signaling pathway genes (24 genes, corrected p-value = 8.90E-06).

### Co-expression modules in adipose are associated with S_I_ and related metabolic phenotypes in Caucasians

**Figure 3:**
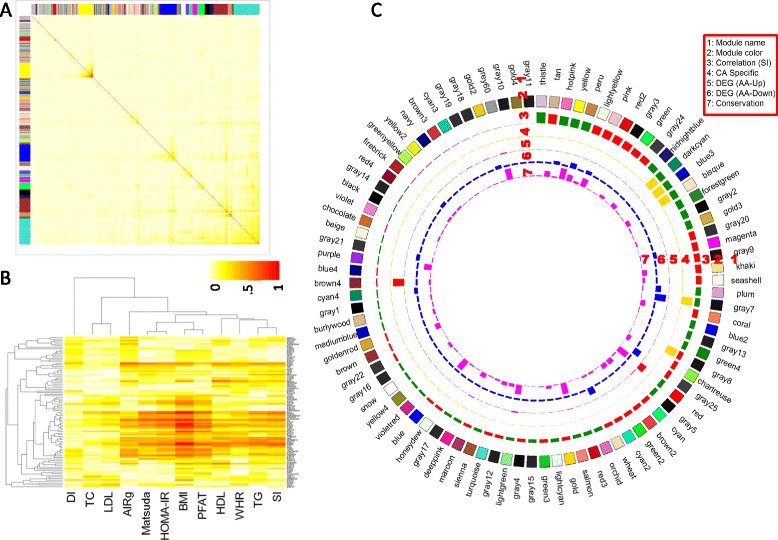
**Weighted gene co-expression analysis of adipose tissue transcript in adipose tissue from Caucasian (CA) subjects identified gene networks (modules) associated with gluco-metabolic phenotypes. A)** Topological Overlap Matrix (TOM) plot of the adipose tissue expression network connections in 99 CA subjects. Light color represents low topological overlap and progressively darker red color represents higher overlap. Each module is assigned by unique color; these are shown along the left side and the top, **B)** Correlation heatmap of 93 module eigengenes and 12 gluco-metabolic traits, **C)** A Circos plot showing module names (Ring 1), module color representation (Ring 2), correlations between module eigengenes and insulin sensitivity (S_I_) (Ring 3), with the bar height representing absolute value of correlation coefficient (range = [0.0012,0.53]) and the bar color for the correlation sign (red – positive and green – negative), module specificity to CA (high bar – CA-specific and low bar – not CA-specific) (Ring 4), significance of the enrichment for up-regulated genes in AAs with the bar height representing –log10(FET p value) (Ring 5) (range = [2.2e-39,1]), significance of the enrichment for down-regulated genes in AAs with the bar height representing –log10(FET p value) (Ring 6) (range = [5.8e-6,1]), and significance of the best enrichment for the modules in AA with the bar height representing –log10(FET p value) (Ring 7) (range = [0, 0.015]). The module names and color representations match those in Figure 3A. The modules are sorted based on the correlations between the module eigengenes and S_I_.

Network distance coupled with hierarchical clustering and dynamic tree cutting methods implemented in WGCNA identified 93 co-expression networks (modules) in adipose tissue of CA subjects (module sizes from 1,957 to 10 transcripts). Modules are denoted by colors (Figure 3A). Four modules in CA subjects included >1,000 probes, the largest being the “turquoise” module, with 1,957 transcript probes. Transcripts that could not be included in any module (3,777 of 17,434 probes) were classified as “gray”. Module membership of each transcript, simple adjacency matrix-based connectivity, and TOM-based connectivity measures of those transcripts within each module are shown in Additional file 1: Table S4.

Correlation analysis of module eigengenes (principal components of the module) with gluco- and cardio-metabolic traits of CA subjects identified some strong module-trait correlations. As shown in Figure 3B, some modules were correlated with multiple phenotypes (also see Additional file 1: Table S5). The clustering of S_I_ and triglyceride (TG) indicates a significant overlap of modules correlated with these two traits in CA subjects. Modules showing significant positive correlation with S_I_ showed significant negative correlation with TG in CA subjects. Module eigengene of 27 modules showed significant correlation with S_I_ (p ≤ 0.05 and FDR ≤ 0.05) (Figure 3C). Eight large modules (>25 gene members) showed strong correlation (positive or negative correlation ≥0.4) with either FSIGT-derived (S_I_ and AIRg) [26] or OGTT-derived (HOMA-IR and Matsuda index) [25] glucose homeostasis traits (Table 2). The “yellow” module showed strong negative correlation (r = −0.426, P = 4.95E-04) with S_I_ and was strongly enriched for genes involved in inflammatory response (corrected p = 1.7E-26). The *TYROBP* gene (TYRO protein tyrosine kinase binding protein) was the most connected hub gene of this co-expression network module based on TOM-based intramodular connectivity, consistent with the finding of the prominent role of *TYROBP* in other complex diseases [16,31].

**Table 2:** **Adipose tissue co-expression modules associated with insulin sensitivity (S**_**I**_ **) or other glucose homeostasis traits in Caucasians**

**Module (Module size)**		**S**_**I**_	Hub gene* (r, P)	**Module genes** ^**#**^ **associated with S**_**I**_ **(r)**	**AIRg**	**HOMA-IR**	**Matsuda Index**	**BMI**	**% Fat mass**	**TG**	**HDL**	**GO Gene Category Overlap, P, Corrected-P**
Tan (268)	r	0.463	*ANG* (0.35, 0.0004)	*PFKFB3*(0.53), *LOC647276*(0.52)	−0.471	−0.572	0.525	−0.677	−0.523	−0.42	0.378	Enoyl-CoA hydratase activity
	p	1.02E-04			6.92E-05	0.00243	1.33E-09	6.7E-13	4.1E-08	0.045	0.000132	3, 0.000046,NS
Thistle (88)	r	−0.531	*ITGB5* (−0.41, <0.00001)	*PLAC9*(−0.46), *ITGB5*(−0.44)	0.307	0.493	−0.459	0.662	0.522	0.467	−0.44	Cellular defense response
	p	2.9E-06			0.00323	0.022	8.70E-07	1.7E-12	5E-08	0.003	0.0000328	6, 0.000039,NS
Yellow (1027)	r	−0.426	*TYROBP* (−0.37, 0.0002)	*KIAA1598*(−0.53), *ITGAV*(−0.52)	0.286	0.384	−0.364	0.607	0.414	0.44	−0.414	Inflammatory response
	p	4.95E-04			0.0493	0.113	0.000674	4E-09	3.72E-04	0.047	0.00045	93, 3.2E-30,1.7E-26
Hot pink (89)	r	−0.438	*PTPN11* (−0.37, 0.0002)	*TMEM54*(−0.44), *SLC46A3*(−0.4)	0.247	0.514	−0.444	0.593	0.436	0.421	−0.231	Substrate-bound cell migration
	p	3.90E-04			0.00786	0.0353	7.82E-06	9.1E-08	6E-06	0.081	0.00741	4, 0.00025,NS
Peru (167)	r	−0.423	*NQO1* (−0.43, <0.00001)	*GNG2*(−0.53), *UCHL1*(−0.5)	0.198	0.475	−0.405	0.683	0.511	0.364	−0.262	Steroid metabolism
	p	6.02E-04			0.0341	0.055	7.13E-05	1.5E-12	2E-08	0.038	0.00662	10, 0.0000059,0.031
Pink (359)	r	0.411	*SDHB* (0.27, 0.0073)	*AZGP1*(0.5), *CKB*(0.49)	−0.306	−0.353	0.344	−0.491	−0.362	−0.36	0.418	Mitochondrion
	p	3.53E-04			0.024	0.161	0.000372	9.7E-07	0.0002	0.032	0.00133	87, 3.6E-59,1.9E-55
Forest green (118)	r	−0.321	*LOC728973* (0.30, 0.0022)	*SNCG*(−0.35), *CES2*(−0.35)	0.272	0.418	−0.42	0.455	0.29	0.311	−0.182	Telomerase holoenzyme complex
	p	0.0691			0.0535	0.198	0.00476	0.00222	0.0104	0.434	0.102	2, 0.00024,NS
Light yellow (186)	r	0.413	*DCI* (0.39, <0.00001)	*ARHGEF16*(0.44), *HADH*(0.43)	−0.306	−0.336	0.323	−0.409	−0.269	−0.38	0.344	Mitochondrion
	p	7.3E-06			0.00765	0.0767	2.68E-06	1.6E-05	0.00163	0.038	0.00238	27, 1.1E-11, 5.8E-08

Large co-expression modules (>25 gene member) showing strong significant correlation (r > + or – 0.4, p <0.05 and FDR < 0.05) with insulin sensitivity (S_I_) or other OGTT- and FSIVGT-derived glucose homeostasis traits in Caucasians are shown. Correlation and significance of each module eigengenes with glucose homeostasis and other metabolic traits are shown. *gene showing highest TOM-based intramodular connectivity (to.in.norm), ^#^top selected S_I-_associated genes shown (if direction matched with eigengene).

The “pink” and “light yellow” modules, which are both enriched for mitochondrion genes are positively correlated with S_I_ with r = 0.411 (P = 3.53E-04) and 0.413 (P = 7.3E-06), respectively. Two nuclear-encoded mitochondrial genes, *SDHB* (Succinate dehydrogenase complex, subunit B) and *DCI* (Dodecenoyl-Coenzyme A delta isomerase or ECI1), were the hub genes for these two modules. The “light yellow” module included genes involved in carboxylic acid metabolism (corrected p = 5.7E-06), while the “pink” module was enriched for branched-chain amino acid (valine, leucine, and isoleucine) degradation genes (corrected p = 2.70E-14, see Additional file 1: Table S6).

### Co-expression modules in adipose are associated with S_I_ and related metabolic phenotypes in African Americans

**Figure 4:**
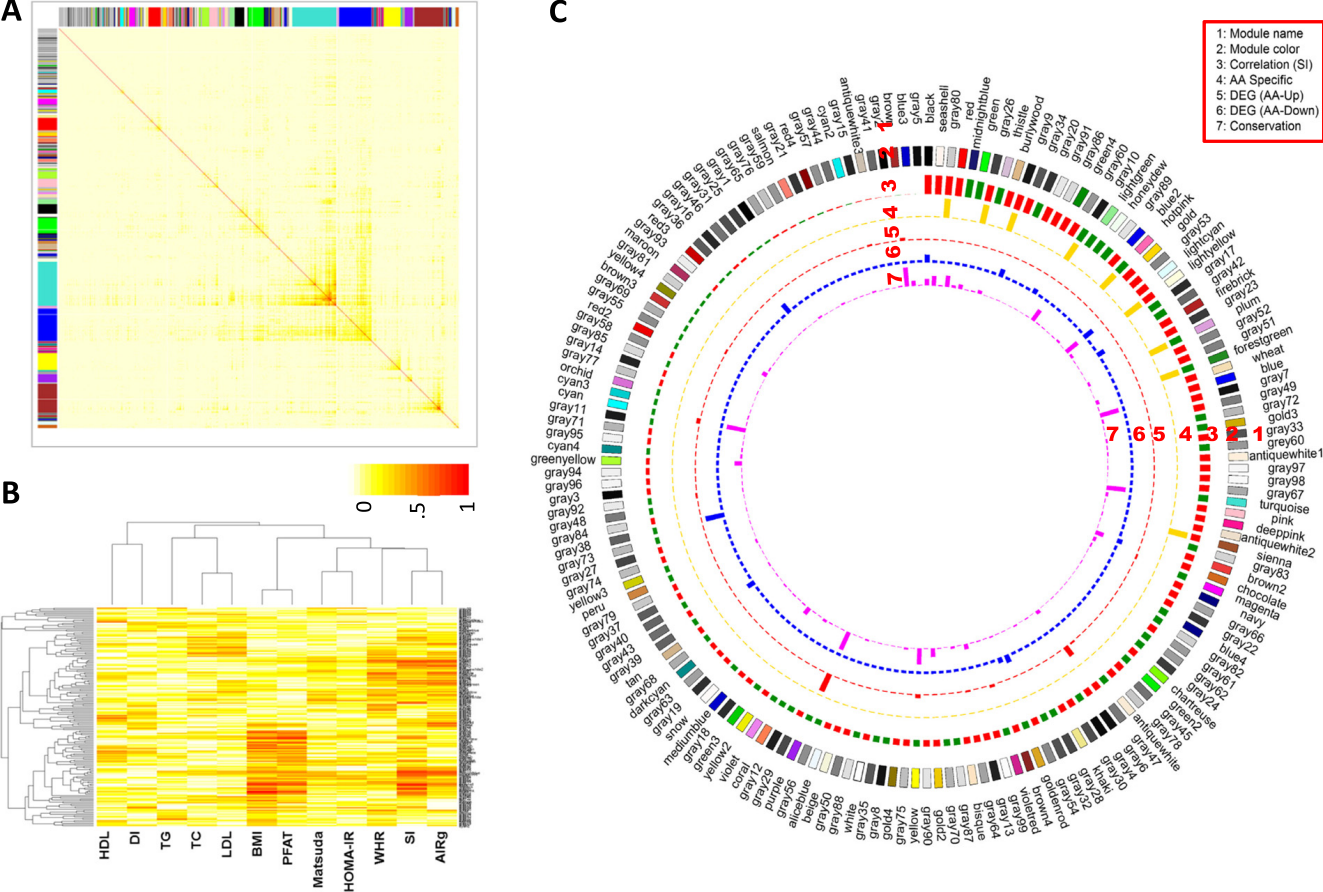
**Weighted gene co-expression analysis of adipose tissue transcript in adipose tissue identified distinct pattern of association between gene networks (modules) and gluco-metabolic phenotypes in African American subjects. A)** TOM plot of the adipose tissue indicates distinct expression network connections in 37 AA subjects. Light color represents low topological overlap and progressively darker red color represents higher overlap. Each module is assigned by unique color; these are shown along the left side and the top, **B)** Correlation heatmap of 173 module eigengenes and 12 gluco-metabolic traits, **C)** A Circos plot showing module names (Ring 1), module color representation (Ring 2), correlations between module eigengenes and insulin sensitivity (S_I_) (Ring 3), with the bar height representing absolute value of correlation coefficient (range = [0.0033,0.61]) and the bar color for the correlation sign (red – positive and green – negative), module specificity to AA (high bar – AA-specific and low bar – not AA-specific) (Ring 4), significance of the enrichment for up-regulated genes in AAs with the bar height representing–log10(FET p value) (Ring 5) (range = [8.2e-19,1]), significance of the enrichment for down-regulated genes in AAs with the bar height representing –log10(FET p value) (Ring 6) (range = [1.8e-7,1]), and significance of the best enrichment for the modules in CA with the bar height representing–log10(FET p value) (Ring 7) (range = [0, 0.13]). The module names and color representations match those in Figure 4A. The modules are sorted based on the correlations between the module eigengenes and S_I_.

**Figure 5:**
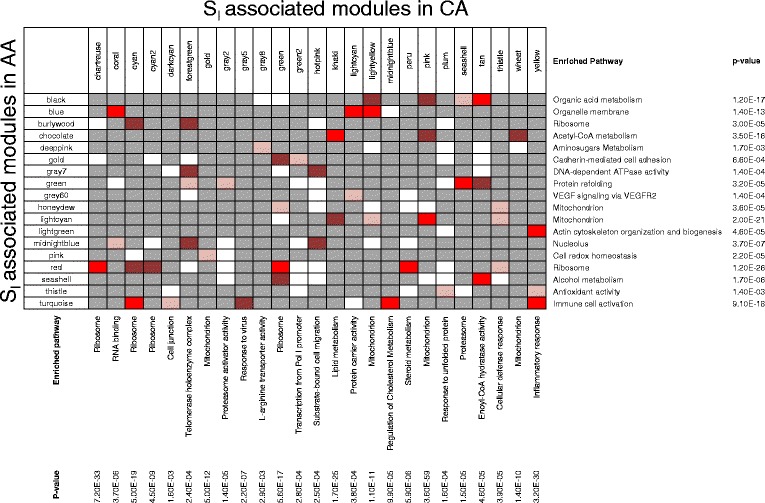
**Overlap of insulin sensitivity (S**_**I**_ **) associated modules in Caucasians and African Americans.** Fisher’s exact test (FET) analysis was used to identify overlap among gene members of S_I_-associated modules in CA and AA subjects. S_I_-associated modules in CAs that showed significant overlap (FET_P value < 3.1E-06) with at least one S_I_-associated module in AAs, or vice versa, are shown. P ≥ 0.05, P ≤ 3.1E-06, P ≤ 1.0E-10 and P ≤ 1.0E-20 are marked in gray, pink, brown, and red, respectively. Enrichment of these modules for biological pathways or processes is shown.

WGCNA of adipose tissue transcripts of 37 African American subjects using the same parameters as described above for Caucasians (beta = 5, scale R^2 = 0.81, trunc. R^2 = 0.99, hiercutoff =0.99 and minModuleSize =10, Additional file 2: Figure S1) identified 173 co-expression modules (module size ranges from 1980 to 10 probes, Figure 4A, Additional file 1: Table S7). Three modules in AA subjects included >1,000 probes, the largest being the “turquoise” module, with 1980 transcript probes. In AA subjects, clustering of module-trait correlations indicated the strongest overlap between S_I_- and AIRg-associated modules (Figure 4B). S_I_ was correlated with the eigengene of 34 modules (p ≤ 0.05 and FDR ≤ 0.05) (Figure 4C and Additional file 1: Table S8). Sixteen large modules showed strong correlations (positive or negative correlation ≥0.4) with either FSIGT- or OGTT-derived glucose homeostasis traits in AA subjects (Table 3). Those with the strongest negative correlations with S_I_ were “midnight blue” (r = −0.58, p = 4.050E-04), “green” (r = −0.56, p = 0.001), and “thistle” (r = −0.52, p = 0.015). These modules were enriched for genes involved in function of nucleolus (p = 3.7E-07), protein refolding (p = 0.000032), and antioxidant activity (p = 0.0014), respectively (Additional file 1: Table S9). Annexin A1 (*ANXA1* or Lipocortin I), a gene with phospholipase A2 inhibitory activity that belongs to a family of Ca(2+)-dependent phospholipid binding proteins, showed a strong negative correlation with insulin sensitivity (r = −0.66, p <0.00001) and the hub gene of the “midnight blue” module. The “black” module showed the strongest positive correlation with S_I_ (r = 0.61, p = 0.001) in adipose tissue of AA subjects. This module showed significant overlap (p ≤ 3.1E-06) with “tan” (57 genes), “light yellow” (33 genes), and “pink” (41genes) modules identified in CA subjects (Figure 5). Similar to the “light yellow” module in CA subjects, the “black” module in AA subjects was enriched for genes involved in mitochondrial carboxylic acid metabolism (corrected p = 2.80E-13); the nuclear encoded mitochondrial gene *DCI* appeared as the hub gene for this module.

**Table 3:** **Adipose tissue co-expression modules associated with insulin sensitivity (S**_**I**_ **) or other glucose homeostasis traits in African Americans**

**Module (Module size)**		**S**_**I**_	Hub gene* (r, P)	**Module genes# associated with S**_**I**_ **(r)**	**AIRg**	**HOMA-IR**	**Matsuda Index**	**BMI**	**PFAT**	**TG**	**HDL**	**GO Gene Category Overlap, P, Corrected-P**
Black (441)	r	0.61	*DCI*	*BCKDHA* (0.7)	−0.39	−0.29	0.32	−0.52	−0.47	−0.05	0.01	Organic acid metabolism
	p	0.001	(0.56,0.0003)		0.012	0.389	0.117	0.005	0.002	0.933	0.829	46, 1.2E-17,6.5E-14
Blue2 (39)	r	0.42	*LOC392871*	*LOC340274* (0.44)	−0.27	−0.22	0.09	−0.71	−0.56	−0.06	0.24	Translational elongation
	p	0.007	(0.44,0.0066)		0.017	0.472	0.241	9.530E-06	3.280E-04	0.594	0.226	2, 0.00034,NS
Brown2 (51)	r	0.29	*NPLOC4*		−0.41	−0.16	0.17	−0.20	−0.19	−0.03	−0.18	FGFR signaling pathway
	p	0.271	(−0.4,0.0132)		0.027	0.030	0.133	0.165	0.260	0.837	0.307	2, 0.0026,NS
Burlywood (54)	r	0.50	*LOC646672*	*THYN1* (0.55)	−0.49	−0.12	0.30	−0.28	−0.29	−0.19	0.03	Ribosome
	p	0.013	(0.12,0.4803)		0.010	0.584	0.008	0.078	0.118	0.913	0.556	3, 0.00003,NS
Chartreuse (67)	r	0.26	*DTWD1*		−0.45	−0.04	0.10	0.03	0.00	−0.14	−0.27	Protein deubiquitination
	p	0.109	(0.003,0.9878)		0.009	0.188	0.134	0.850	0.836	0.971	0.166	3, 0.00022,NS
Forest green (115)	r	−0.38	*DCTN1*	*PCNXL3* (−0.48)	0.44	0.04	−0.10	0.15	0.03	−0.06	0.02	Drug transport
	p	0.049	(−0.47,0.0031)		0.025	0.045	0.597	0.345	0.814	0.361	0.964	3, 0.00075,NS
Gold (147)	r	0.41	*THYN1*	*LOC651198* (0.63)	−0.35	−0.26	0.19	−0.49	−0.47	−0.12	0.15	Cadherin-mediated cell adhesion
	p	0.018	(0.52,0.0011)		0.019	0.212	0.005	0.001	0.007	0.728	0.774	4, 0.00066,NS
Green (618)	r	−0.56	*FAM100A*	*AHCY* (−0.72)	0.54	0.26	−0.36	0.27	0.29	0.14	0.02	Protein refolding
	p	0.001	(−0.46,0.0043)		0.001	0.039	0.043	0.132	0.051	0.607	1.000	6, 0.000032,NS
Honeydew (69)	r	−0.43	*C4orf18*	*VGLL3* (−0.56)	0.17	0.20	−0.24	0.58	0.65	0.24	−0.05	Mitochondrion
	p	0.012	(−0.31,0.0659)		0.141	0.898	0.135	1.220E-04	4.320E-05	0.523	0.554	9, 0.000036,NS
Hot pink (93)	r	−0.42	*MBD3*	*PIP5K1C* (−0.55)	0.47	0.16	−0.25	0.21	0.19	0.06	0.00	Intracellular transport
	p	0.034	(−0.5,0.0016)		0.019	0.025	0.131	0.213	0.252	0.649	0.803	15, 1.5E-06,0.0077
Light green (164)	r	−0.43	*ZYX*	*MYO9B* (−0.63)	0.21	0.31	−0.31	0.39	0.42	0.13	−0.34	Actin cytoskeleton organization and biogenesis
	p	0.011	(−0.52,0.0009)		0.104	0.784	0.075	0.059	0.052	0.978	0.047	11, 0.000046,NS
Magenta (287)	r	−0.29	*DPP3*	*TUBB2A* (−0.57)	0.51	0.16	−0.22	0.16	0.22	0.12	0.22	Unsaturated fatty acid biosynthesis
	p	0.124	(−0.32,0.0559)		0.006	0.034	0.081	0.617	0.212	0.718	0.211	4, 7.1E-07,0.0037
Midnight blue (186)	r	−0.58	*ANXA1*	*SYNM* (−0.71)	0.54	0.23	−0.34	0.41	0.42	0.26	−0.11	Nucleolus
	p	4.050E-04	(−0.66,<0.00001)		3.870E-04	0.178	0.071	0.011	0.010	0.907	0.319	11, 3.7E-07,0019
Red (464)	r	0.58	*LOC644315*	*LOC401676* (0.58)	−0.51	−0.26	0.28	−0.42	−0.39	−0.31	0.18	Ribosome
	p	0.005	(0.41,0.0113)		0.003	0.250	0.045	0.007	0.012	0.632	0.536	24, 1.2E-26,6E-23
Seashell (124)	r	0.60	*MOCS1*	*CNNM3* (0.62)	−0.46	−0.27	0.23	−0.61	−0.54	−0.06	−0.01	Alcohol metabolism
	p	0.004	(0.52,0.001)		0.003	0.191	0.044	6.380E-05	4.050E-04	0.817	0.734	10, 1.7E-06,0.0088
Thistle (92)	r	−0.52	*C1GALT1C1*	*RNF145* (−0.54)	0.45	0.28	−0.33	0.22	0.35	0.16	0.02	Antioxidant activity
	p	0.015	(−0.49,0.002)		0.072	0.262	0.024	0.182	0.009	0.576	0.823	3, 0.0014,NS

Large co-expression modules (>25 gene member) showing strong significant correlation (r > + or – 0.4, p <0.05 and FDR < 0.05) with insulin sensitivity (S_I_) or other OGTT- and FSIVGT-derived glucose homeostasis traits in African Americans are shown. Correlation and significance of each module eigengenes with glucose homeostasis and other metabolic traits are shown. *gene showing highest TOM-based intramodular connectivity (to.in.norm), ^#^top selected S_I_-associated genes shown (if direction matched with eigengene).

To validate our findings, we compared our co-expression modules with the coexpression network modules from an independent human adipose gene expression data (called deCODE data) from 640 Caucasian subjects [23]. Twelve modules in the AA network and nineteen modules in the CA network significantly (Bonferroni corrected FET p-value < 0.05) overlap with the network modules from the deCODE data. Notably, 5 of the top 10 CA modules associated with S_I_ identified in our study significantly overlap with the modules from the deCODE data, and the yellow (inflammatory response), pink (mitochondrion) and peru (steroid metabolism) modules are among the top five most conserved modules, with FET p values as 3.6 × 10^−151^ (5.6 fold enrichment), 6.6 × 10^−58^ (17.5 fold enrichment) and 1.4 × 10^−28^ (13 fold enrichment), respectively. On the other hand, 4 of the top 10 AA modules associated with S_I_ significantly overlapped with the gene modules from the deCODE data, but to a lesser degree (Bonferroni corrected FET p-values are between 8.2 × 10^−3^ and 2.4 × 10^−8^). This comparison basically validates our findings about both the AA and CA subjects, although the results from the CA data are more profoundly replicated in another independent study in CA subjects. However, clinical and metabolic phenotype data are not publicly available for deCODE study subjects to validate our other findings, including correlation between modules and phenotypes.

### Some metabolic trait-associated modules show ethnic differences

We tested the enrichment of modules for genes differentially expressed in AA compared to CA subjects. Nineteen co-expression modules in adipose of CA subjects were enriched for differentially expressed genes (enrichment p ≤0.05). Among these modules, five showed significant correlations with S_I_ (Additional file 1: Table S10A). The “red” module in CA subjects included the largest number of genes (50 genes, p =3.17E-26) upregulated in African Americans, correlated with AIR_G_ (r = 0.276, p = 0.0476), and was enriched for genes involved in cytoskeletal protein binding and adherens junction (enrichment p-value <3.0E-10). Similarly, 18 transcript modules in adipose of AA subjects were enriched for genes differentially expressed in African Americans compared to Caucasians, and five were significantly correlated with S_I_ (Additional file 1: Table S10B). The “magenta” network module in AA adipose tissue was enriched for genes differentially up-regulated in AA compared to CA subjects (p = 0.0084, includes 11 up-regulated transcripts), showed a significant positive correlation with AIR_G_ (r = 0.51; p = 0.006), and was enriched for genes involved in unsaturated fatty acid biosynthesis (corrected p = 0.0037) and glucose metabolism (corrected p = 0.007).

We used the modular differential connectivity (MDC) metric [16] to quantify differences in adipose tissue transcript network organization between CA and AA subjects. This analysis suggested significant differential connectivity (FDR < 0.2) in 59 CA adipose tissue-derived modules (Additional file 1: Table S11), almost all showing gain of connectivity (MDC > 1). Among these modules, 13 showed significant correlation with S_I_ in CA subjects and were enriched for genes in functional categories important for insulin resistance (Table 4). Similar analyses of AA adipose tissue-derived networks identified MDC in 11 modules; 10 had high gain of connectivity (MDC> > 1) and 1 had loss of connectivity (MDC < 1). Only one of these modules was correlated with insulin sensitivity (Additional file 1: Table S12).

**Table 4:** **Insulin sensitivity associated co-expression modules in adipose tissue of Caucasian subjects shows differential connectivity**

**Module (Module size)**	**MDC**	**MDC FDR**	**AA.DE Overlap (P-value)**	**S**_**I**_ **correlation (p-value)**	**BMI correlation (p-value)**	**GO Gene Category Overlap, p-value**
Bisque (121)	11.16	0	3	−0.323	0.357	Response to virus
			(NS)	(0.04)	(0.11)	23, 6.70E-26
Green (487)	4.34	0	9	0.337	−0.593	Ribosome
			(NS)	(0.01)	(1.35E-06)	27, 5.60E-17
Khaki (134)	4.13	0	4	0.284	−0.322	Lipid metabolism
			(NS)	(7.48E-04)	(5.24E-03)	39, 1.7E-25
Pink (359)	3.02	0	15	0.411	−0.491	Mitochondrion
			(NS)	(3.53E-04)	(9.73E-07)	87, 3.6E-59
Gray7 (15)	2.8	0.02	5	−0.264	0.458	Adherens junction
			(1.03E-05)	(0.01)	(1.34E-06)	2, 0.00014
Green4 (22)	2.37	0.04	0	−0.233	0.378	IL4 - antiapoptotic action
			(NS)	(0.04)	(2.23E-04)	2, 0.00034
Red2 (43)	2.03	0	0	0.385	−0.34	Mitochondrion
			(NS)	(7.72E-05)	(5.42E-04)	6, 0.0011
Wheat (157)	2.02	0	12	0.192	−0.101	Mitochondrion
			(6.36E-03)	(0.03)	(0.20)	21, 1.40E-10
Gray3 (16)	1.91	0.04	1	0.38	−0.392	Guanylate kinase activity
			(NS)	(4.42E-04)	(1.09E-05)	2, 2.60E-05
Peru (167)	1.46	0	10	−0.423	0.683	Steroid metabolism
			(0.05)	(6.02E-04)	(1.45E-12)	10, 5.90E-06
Thistle (88)	1.23	0.04	4	−0.531	0.662	Cellular defense response
			(NS)	(2.89E-06)	(1.67E-12)	6, 3.90E-05
Tan (268)	1.11	0	4	0.463	−0.677	Enoyl-CoA hydratase activity
			(NS)	(1.02E-04)	(6.73E-13)	3, 4.60E-05
Light yellow (186)	1	0.04	2	0.413	−0.409	Mitochondrion
			(NS)	(7.31E-06)	(1.59E-05)	27, 1.1E-11

MDC, modular differential connectivity; FDR, false discovery rate; S_I_, insulin sensitivity derived from MINMOD analysis of FSIVGT; AA.DE, genes differentially expressed in AA compared to CA subjects.

We identified 16 and 15 conserved S_I_-associated network modules in CAs and AAs, respectively (FDR ≤ 0.05) (Table 5A and B, Figures 3C and 4C). These modules showed significant overlap (FET_p value ≤3.1E-06) of module gene members in both ethnicities, and were significantly enriched for genes correlated with S_I_ in both ethnicities. We also identified five and nine ethnic-specific S_I_-associated network modules, respectively, in CAs and AAs (FDR ≤ 0.05) (Table 6). These modules were significantly enriched for genes correlated with S_I_ in only one ethnicity (Figures 3C and 4C). A subset of modules (three in CA and five in AA) also showed no significant overlap (FET_p value > 3.1E-06) of gene members between ethnicities, which indicates the presence of highly ethnic-specific mechanisms associated with insulin sensitivity.

**Table 5:** **Insulin sensitivity associated co-expression modules conserved in adipose tissue of both Caucasian and African American subjects**

**A. Conserved co-expression modules in CA subjects**									
**CA Module (Module Size)**	**S**_**I**_ **correlation (p; FDR)**	**FET p-value (Most Conserved w/ AA Module)**	**Enrichment for S**_**I**_ **correlated genes (FET_P value)**						**GO Gene.Category (Module Overlap; FET_P)**
			**All.corNeg**	**All.corPos**	**CA.corNeg**	**CA.corPos**	**AA.corNeg**	**AA.corPos**	
Blue2 (32)	−0.248 (2.15E-02, 1.24E-03)	2.99E-07 (yellow)	8.30E-05	1.00	1.06E-02	1.00	1.00	1.00	localization of cell (7; 5.90E-05)
Chartreuse (65)	0.221 (8.68E-02; 4.74E-03)	3.05E-38 (red)	1.00	5.68E-10	1.00	6.05E-03	1.00	4.47E-04	Ribosome (17; 7.20E-33)
Coral (183)	0.259 (9.25E-03;6.84E-04)	1.00E-44 (blue)	0.09	4.48E-05	0.96	4.13E-03	0.18	1.44E-05	RNA binding (16; 3.70E-06)
forest green (118)	−0.321 (6.91E-02; 1.38E-05)	2.55E-12 (burlywood)	0.10	1.12E-07	0.83	0.38	5.80E-08	0.17	telomerase holoenzyme complex (2; 2.40E-04)
Green (487)	0.337 (8.79E-03; 4.34E-06)	1.70E-27 (red)	0.75	1.05E-22	0.97	8.29E-24	1.00	1.08E-05	Ribosome (27; 5.60E-17)
Hot pink (89)	−0.438 (3.90E-04; 6.75E-10)	5.74E-13 (midnightblue)	3.52E-26	0.12	4.48E-02	0.75	2.70E-03	0.97	substrate-bound cell migration (4; 2.50E-04)
Khaki (134)	0.284 (7.48E-04; 1.59E-04)	6.96E-45 (chocolate)	1.00	1.66E-04	1.00	3.00E-16	1.00	0.83	lipid metabolism (39; 1.70E-25)
Light yellow (186)	0.413 (7.31E-06; 7.49E-09)	2.54E-20 (blue)	1.35E-02	7.30E-51	0.14	1.15E-09	1.00	0.69	Mitochondrion (27; 1.10E-11)
Peru (167)	−0.423 (6.02E-04; 2.91E-09)	2.92E-60 (red)	1.55E-46	1.00	3.53E-20	1.00	0.89	1.00	steroid metabolism (10; 5.90E-06)
Pink (359)	0.411 (3.53E-04; 9.01E-09)	9.87E-76 (lightcyan)	1.00	1.24E-45	1.00	3.90E-72	1.00	0.97	Mitochondrion (87; 3.60E-59)
Plum (96)	−0.264 (5.34E-02; 5.17E-04)	1.44E-11 (peru)	1.39E-08	0.94	7.61E-03	0.81	0.76	0.84	response to unfolded protein (4; 1.60E-04)
Red (432)	−0.218 (9.92E-01; 5.45E-03)	1.24E-235 (purple)	5.53E-04	1.00	7.56E-04	0.95	1.35E-04	1.00	cytoskeletal protein binding (40; 3.10E-15)
Seashell (135)	0.27 (1.53E-02; 3.66E-04)	4.64E-25 (green)	4.17E-03	1.15E-06	1.00	1.42E-02	0.98	5.34E-13	Proteasome (4; 1.50E-05)
Tan (268)	0.463 (1.02E-04; 5.21E-11)	6.18E-52 (seashell)	4.58E-02	3.32E-88	1.00	3.59E-18	1.00	0.55	enoyl-CoA hydratase activity (3; 4.60E-05)
Thistle (88)	−0.531 (2.89E-06; 2.22E-14)	1.22E-09 (red)	4.30E-10	2.56E-03	4.16E-02	7.11E-04	0.95	0.62	cellular defense response (6; 3.90E-05)
Yellow (1027)	−0.426 (4.95E-04; 2.18E-09)	5.01E-169 (turquoise)	8.05E-105	1.00	3.35E-167	1.00	1.00	1.00	inflammatory response (93; 3.20E-30)
**B. Conserved co-expression modules in AA subjects**									
**AA Module (Module Size)**	**S**_**I**_ **correlation(p; FDR)**	**FET p-value (Most Conserved w/ CA Module)**	**Enrichment for S**_**I**_ **correlated genes (FET_P value)**						**GO Gene.Category (Module Overlap; FET_P)**
			**All.corNeg**	**All.corPos**	**CA.corNeg**	**CA.corPos**	**AA.corNeg**	**AA.corPos**	
Black (441)	0.609 (7.46E-04; 8.07E-07)	2.25E-36 (tan)	0.18	2.28E-101	1.00	1.25E-06	0.86	7.31E-14	organic acid metabolism (46; 1.20E-17)
Blue (1392)	0.359 (5.61E-02; 9.69E-03)	0 (blue)	0.56	6.30E-09	1.00	0.17	1.96E-03	8.27E-13	organelle membrane (93; 1.40E-13)
Burlywood (54)	0.502 (1.25E-02; 9.04E-05)	8.94E-19 (cyan)	0.72	1.73E-09	0.93	3.04E-02	0.95	1.28E-03	Ribosome (3; 3.00E-05)
Chocolate (133)	0.291 (5.73E-02; 4.75E-02)	6.96E-45 (khaki)	1.00	1.52E-04	1.00	5.72E-09	1.00	0.82	acetyl-CoA metabolism (10; 3.50E-16)
Gold (147)	0.41 (1.75E-02; 2.26E-03)	1.96E-11 (green)	1.00	1.70E-04	1.00	1.71E-02	0.96	2.29E-06	Cadherin-mediated cell adhesion (4; 6.60E-04)
Gray7 (26)	0.357 (6.88E-02; 1.02E-02)	4.32E-11 (hotpink)	1.00	8.61E-09	0.88	8.20E-04	0.76	0.80	DNA-dependent ATPase activity (2; 1.40E-04)
Green (618)	−0.562 (9.18E-04; 7.31E-06)	4.64E-25 (seashell)	6.30E-14	1.68E-12	1.00	1.00	6.09E-51	7.30E-29	protein refolding (6; 3.20E-05)
Honeydew (69)	−0.43 (1.23E-02; 1.20E-03)	3.05E-06 (green)	7.98E-08	1.47E-02	4.19E-02	0.11	0.31	0.40	mitochondrion (9; 3.60E-05)
Lightcyan (183)	0.397 (4.21E-03; 3.35E-03)	9.87E-76 (pink)	0.75	8.41E-29	0.86	7.09E-29	1.00	0.93	mitochondrion (35; 2.00E-21)
Lightgreen (164)	−0.43 (1.06E-02; 1.20E-03)	5.34E-67 (yellow)	4.15E-15	1.00	4.70E-19	1.00	0.17	1.00	actin cytoskeleton organization and biogenesis(11; 4.60E-05)
midnightblue (186)	−0.578 (4.05E-04; 3.53E-06)	5.74E-13 (hotpink)	3.36E-21	2.75E-03	1.00	0.97	6.66E-23	1.94E-05	nucleolus (11; 3.70E-07)
Red (464)	0.578 (4.51E-03; 3.53E-06)	2.92E-60 (peru)	2.37E-36	2.83E-06	0.75	0.99	1.12E-03	1.76E-08	Ribosome (24; 1.20E-26)
Seashell (124)	0.601(3.88E-03; 1.19E-06)	6.18E-52 (tan)	0.13	2.41E-53	1.00	6.97E-06	1.00	1.73E-02	alcohol metabolism (10; 1.70E-06)
Thistle (92)	−0.517(1.54E-02; 4.97E-05)	3.05E-07 (yellow)	8.67E-05	0.26	1.22E-02	0.64	0.06	0.81	antioxidant activity (3; 1.40E-03)
Turquoise (1980)	−0.315(4.74E-01; 2.84E-02)	5.01E-169 (yellow)	4.94E-23	1.00	5.89E-47	1.00	1.00	1.00	immune cell activation (82; 9.10E-18)

All.corNeg/.corPos, Enrichment for genes that are negatively/positively correlated with S_I_ in both AA and CA; CA.corNeg/.corPos; Enrichment for CA-specific genes that are negatively/positively correlated with S_I_; AA.corNeg/.corPos; Enrichment for AA-specific genes that are negatively/positively correlated with S_I_; FDR, False discovery rate; FET, Fisher’s exact test.

**Table 6:** **Ethnicity specific Insulin sensitivity associated co-expression modules**

**Module (Module Size)**	**SI correlation(p; FDR)**	**FET p-value(w/ Most Conserved Module)**	**Enrichment for SI correlated genes(FET_P value)**						**GO Gene.Category (Module Overlap; FET_P)**
			**All.corNeg**	**All.corPos**	**CA.corNeg**	**CA.corPos**	**AA.corNeg**	**AA.corPos**	
**CA specific modules**									
Bisque(121)	−0.323(3.59E-02; 1.20E-05)	4.57E-40	0.67	1.00	1.85E-16	1.00	0.99	0.60	response to virus (23; 6.70E-26)
Blue3(25)	−0.325(5.49E-03; 1.04E-05)	6.16E-06	0.33	1.00	1.02E-07	1.00	1.00	1.00	endoplasmic reticulum (5; 4.40E-04)
Darkcyan(63)	−0.325(1.06E-02; 1.04E-05)	1.24E-09	0.57	0.95	8.09E-05	0.77	0.66	0.53	cell junction (4; 1.60E-03)
Gray7(15)	−0.264(6.88E-03; 5.17E-04)	0.000124	1.00	1.00	5.46E-06	1.00	1.00	1.00	adherens junction (2; 1.40E-04)
Gray8(14)	0.228(3.32E-03; 3.41E-03)	1.67E-05	1.00	1.00	1.00	5.04E-05	1.00	0.58	L-arginine transporter activity (1; 2.90E-03)
**AA specific modules**									
Forest green(115)	−0.38(4.94E-02; 5.47E-03)	3.68E-18	0.79	0.79	1.00	0.84	1.05E-05	0.39	drug transport (3; 7.50E-04)
Gray10(24)	−0.433(6.29E-03; 1.09E-03)	0.000413	0.68	1.00	0.86	1.00	2.21E-05	0.78	vasculogenesis (2; 5.00E-04)
Gray26(20)	0.519(1.77E-03; 4.58E-05)	0.000935	0.62	1.00	1.00	0.82	0.29	1.11E-05	cellular catabolism (5; 5.30E-04)
Gray80(12)	0.6(1.21E-02; 1.25E-06)	9.62E-06	0.44	0.44	1.00	1.00	1.00	6.77E-08	UDP-N-acetylglucosaminebiosynthesis (1; 7.20E-04)
Gray9(25)	0.49(1.14E-03; 1.44E-04)	0.0142	0.33	8.34E-04	0.87	1.00	2.51E-04	0.06	regulation of small GTPasemediated signal transduction (2; 9.80E-04)
Hot pink(93)	−0.415(3.39E-02; 1.93E-03)	2.29E-05	0.14	0.64	0.94	1.00	3.08E-05	0.05	intracellular transport (15; 1.50E-06)
Lightyellow(152)	0.391(2.63E-02; 3.99E-03)	2.67E-15	1.00	0.99	0.98	1.00	3.32E-02	6.95E-09	oxidoreductase activity, acting on superoxideradicals as acceptor (2; 2.90E-04)
Plum(113)	−0.384(2.76E-02; 4.88E-03	5.67E-13	0.90	6.43E-03	0.95	0.83	5.02E-07	0.14	small GTPase mediated signal transduction (10; 1.40E-05)
Sienna(147)	0.299(1.37E-01; 4.02E-02)	1.61E-06	1.00	1.00	1.00	0.96	0.90	1.98E-04	P-P-bond-hydrolysis-driven transporter activity (6; 5.60E-05)

All.corNeg/.corPos, Enrichment for genes that are negatively/positively correlated with S_I_ in both AA and CA subjects; CA.corNeg/.corPos; Enrichment for CA-specific genes that are negatively/positively correlated with S_I_; AA.corNeg/.corPos; Enrichment for AA-specific genes that are negatively/positively correlated with S_I_; FDR, False discovery rate; FET, Fisher’s exact test.

**Figure 6:**
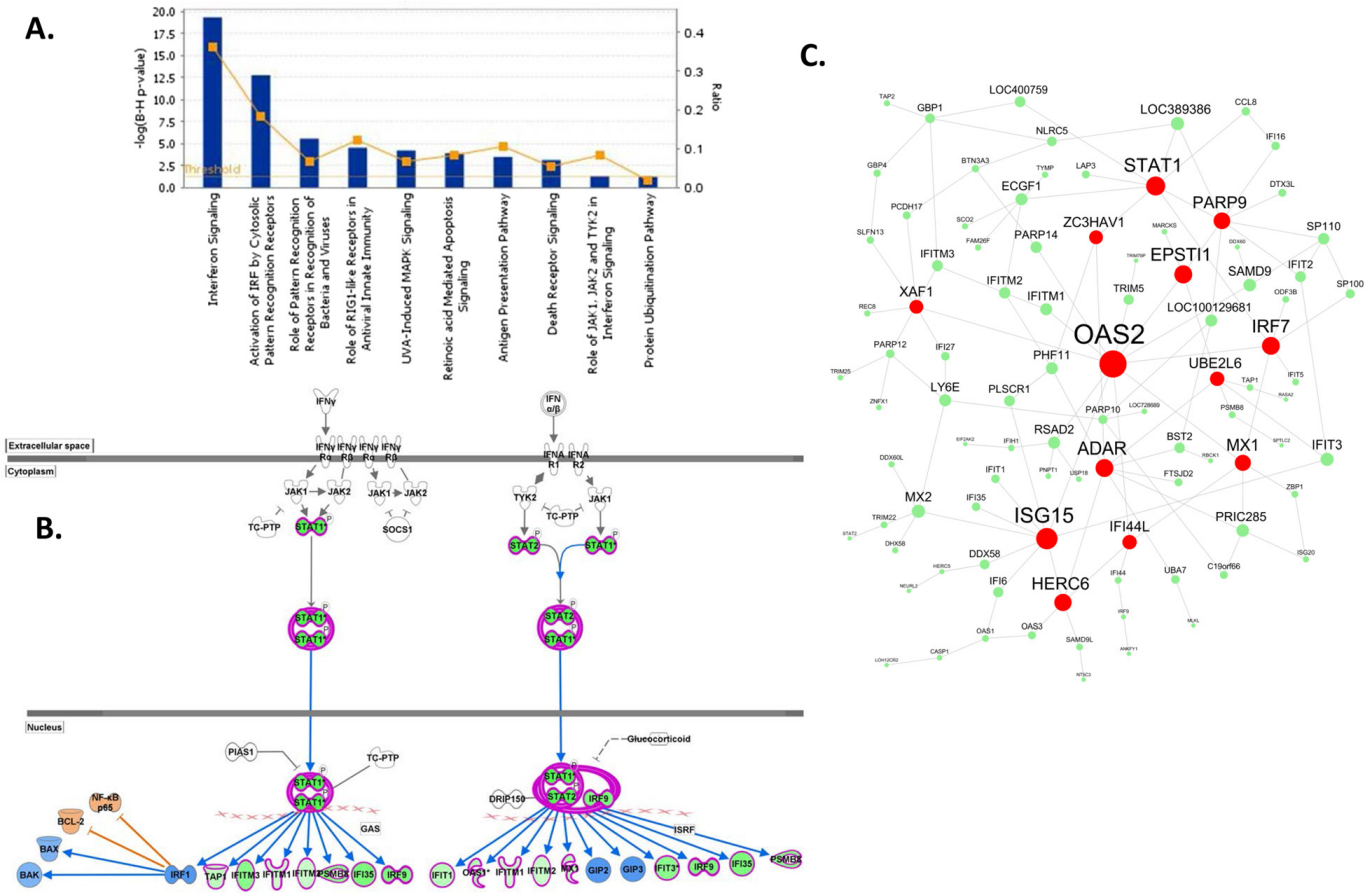
**The “bisque” module is a Caucasian-specific insulin sensitivity (S**_**I**_ **)-associated network module. A)** Enrichment of the “bisque” module genes for canonical pathways based on IPA analysis, **B)** Genes in this module are involved in the interferon signaling pathway, and are negatively correlated with S_I_ (shown in green color), and **C)** Network plots of the “bisque” module in adipose of CA subjects. The red nodes are “drivers”; the size of a node is proportional to the number of nodes in the node's 2-layer neighborhood.

Among these ethnicity-specific S_I_-associated modules, “bisque” is most significantly enriched for transcripts negatively correlated with S_I_ in CAs (p =1.85E-16). This module, which has a strong differential connectivity (MDC = 11.16), is enriched for the interferon signaling pathway (in IPA analysis B-H P-value = 5.01E-20, Figure 6A, B). Several interferon-inducible genes involved in immune response to viral infection (including *OAS2*, *ISG15*, *STAT1,* and *ADAR*) were identified as “drivers” in this sub-network (Figure 6C) based on the ARACNE [29] and key driver analysis [16,31]. The pro-inflammatory T-cell-derived cytokine interferon gamma (IFNγ) promotes the macrophage M1 phenotype. IFNγ also modulates adipocyte metabolic functions. McGillicuddy et al. (2009) demonstrated that IFNγ may play an important role in T-cell modulation of diet-induced obesity, insulin resistance, and type 2 diabetes via activation of the adipocyte JAK-STAT pathway [32]. IFNγ attenuates insulin signaling, lipid storage, and differentiation in human adipocytes, an effect likely mediated via sustained STAT1 activation [33]. Indeed, 19 genes of this module were associated with metabolic and cardiovascular disorders based on the Genetic Association Database (http://geneticassociationdb.nih.gov/). Specifically, *IRF7* and *NLRC5* are associated with type 2 diabetes, and *SP110* and *ZBP1* are associated with waist circumference and body fat distribution, respectively.

**Figure 7:**
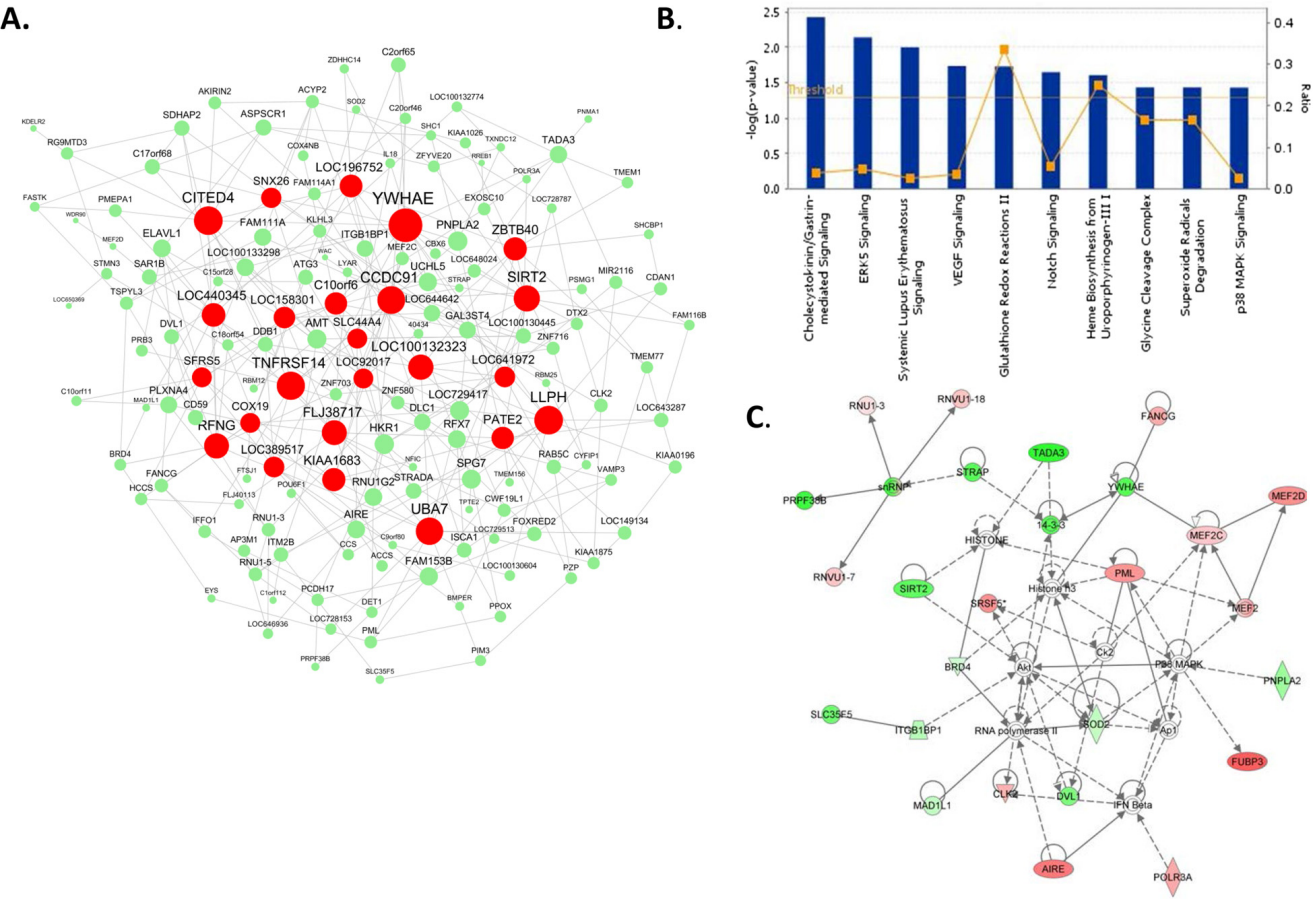
**The “light yellow” module is an African American-specific insulin sensitivity (S**_**I**_ **)-associated network module. A)** Network plots of the “light yellow” module in adipose of AA subjects. The red nodes are “drivers”; the size of a node is proportional to the number of nodes in the node's 2-layer neighborhood, **B)** Enrichment of module genes for canonical pathways in IPA analysis, **C)** The most significant network among the module members based on IPA interaction network analysis involves the YWHAE (14-3-3) gene. Genes positively or negatively correlated with S_I_ in this interaction network are shown in red and green, respectively.

Among the 9 AA-specific and S_I_-associated modules, the “light yellow” module is most enriched for the transcripts positively correlated with S_I_ in AAs (p = 6.95E-09). The top driver gene of this sub-network (Figure 7A), *YWHAE* (14-3-3-epsilon protein), may play a role in the regulation of S_I_ by modulating the interaction between the insulin receptor and IRS1. This module was enriched for oxidoreductase activity (p = 2.90E-04), and was marginally enriched for genes in ERK5 (p = 0.007) and vascular endothelial growth factor (VEGF) signaling pathways (p = 0.018) (Figure 7B) on IPA analysis. Both pathways include the *YWHAE* gene. The top interaction network based on the IPA database also includes YWHAE (14-3-3) (Figure 7C). We identified 35 genes from this module in the genetic association database, including genes associated with type 2 diabetes, obesity, and insulin resistance (i.e. *PNPLA2, SOD2, ZNF716, VAMP3, SHC1, IL18, DLC1, CYFIP1,* and *ASPSCR1*).

Although the “bisque” and “light yellow” modules significantly overlap with others in the AA and CA networks (Table 6), less than 26% of their members are in common with their counterparts. Therefore, they are considered as ethnicity-specific. ARACNE analysis identified seven Caucasian specific driver genes (*ISG15, ADAR, EPSTI1, IRF7, HERC6, MX1 and IFI44L*) for the “bisque” module. Similarly, among the 24 drivers of the “light yellow” module in AA, eight (*YWHAE, LLPH, TNFRSF14, CCDC91, FLJ38717, LOC196752, COX19, and SLC44A4*) are also the drivers of the same set of genes in CA. However, the remaining 16 drivers (*CITED4, UBA7, SIRT2, LOC100132323, RFNG, LOC440345, KIAA1683, ZBTB40, C10orf6, PATE2, LOC158301, LOC389517, LOC641972, SNX26/ARHGAP33, LOC92017, and SFRS5/SRSF5*) of this module appear to be AA-specific.

## Discussion

The primary goal of our study was to measure transcriptional modulation related to S_I_ and related metabolic traits in adipose tissue, and to examine how these might relate to molecular mechanisms of insulin resistance in African Americans compared to European Americans. Our analyses revealed distinct ethnic-specific, S_I_-associated configurations and biological functions of these co-expression modules. Our study provides critical insight into the transcriptional mechanisms and molecular processes underlying insulin sensitivity that could be implicated in T2D onset and may explain differences in T2D prevalence between CA and AA individuals.

We identified significant differential expression of 655 transcript probes between CA and AA subjects, which represent 418 genes with known function. A significant enrichment of these genes in important functional categories, including the glutathione-mediated detoxification and oxidative stress response pathways, was observed. Several isoforms of glutathione S-transferases (*GSTT2, GSTT2B, GSTM3,* and *GSTM5*) showed higher expression, while microsomal glutathione S-transferase 1 (*MGST1*) and superoxide dismutase 2 (*SOD2*) showed lower expression in AA subjects compared to CA subjects. The increased GST transcript expression we observed may indicate a stronger oxidative stress response capacity (in response to environmental factors) [34] in African Americans. However, further molecular genetic studies will be required to define its role in relation to insulin resistance and obesity.

Intriguingly, AA subjects show lower expression of a transcript splice form of *USMG5* (DAPIT). DAPIT is a phylogenetically conserved ATP synthase (mitochondrial and lysosomal) that was down-regulated at the mRNA level in skeletal muscle of streptozotocin (STZ)-treated diabetic rats [35]. Its protein expression was up-regulated in epididymal adipose tissue of STZ-treated mice [36]. However, this gene was not associated with insulin sensitivity or BMI in our study. Compared to CA subjects, AA subjects also show significantly lower expression of the *MARCO* gene, which was positively correlated with BMI in CA subjects. The *MARCO* gene is a scavenger receptor expressed on macrophages and dendritic cells. Macrophages from MARCO-deficient mice exhibited lower interleukin-12 (IL-12) production in responses to stimulation with lipopolysaccharide and interferon-γ. MARCO −/− mice have severely impaired ability to clear bacteria from the lungs and increased mortality during bacterial infection, possibly due to impaired Th1 polarization [37,38]. Further studies will be required to reveal the species-specific and tissue-specific role of the *DAPIT* and *MARCO* genes.

We detected associations between several transcript modules, S_I,_ and related metabolic phenotypes. Analysis to identify enrichment of these trait-associated models for functional categories of genes revealed molecular mechanisms that may cause insulin resistance in both ethnic groups, and indicated some pathophysiological mechanisms that are restricted to one ethnic group. The “black” module in adipose of AA subjects overlapped significantly with the “light yellow” module identified in CA subjects. The *DCI* (or *ECI1*) was the hub gene for both modules. This gene encodes a key mitochondrial enzyme involved in beta-oxidation of unsaturated fatty acids [39]. These modules also showed strong enrichment for genes involved in mitochondrial carboxylic acid and branched chain amino acid metabolism. Positive correlations of these modules enriched for mitochondrial function with insulin sensitivity, and negative correlations with BMI in both AA and CA subjects, indicate that a common molecular mechanism may be involved in obesity-associated insulin resistance.

Previous studies had indicated a role of inflammatory and immune response pathway genes in S_I_ and obesity. However, these studies were restricted to Caucasian subjects. In line with these findings, we identified negative correlations between S_I_ and the “yellow” module in adipose of CA subjects (r = −0.426, p = 4.95E-04) and a strong positive correlation of this module with BMI (r = 0.607, p = 4.03E-09). This module was strongly enriched for genes involved in inflammatory response (corrected p = 1.7E-26). IPA indicated enrichment of genes involved in Fcγ receptor-mediated phagocytosis in macrophages and monocytes (p = 2.51E-12). The *TYROBP* is the hub gene for this transcript network module. This gene encodes a transmembrane signaling polypeptide containing an immunoreceptor tyrosine-based activation motif in its cytoplasmic domain. *TYROBP* is involved in signaling events that mediate crosstalk between dendritic cells and natural killer cells. Recently, it was identified as a central regulator of a gene expression network in brain tissue involved in late-onset Alzheimer’s disease [16,40]. The “yellow” module in CA subjects showed the strongest overlap with the “turquoise” module of AA subjects (452 genes, p = 5.01E-169). This “turquoise” module showed enrichment for immune cell activation and the module eigengene showed significant correlation with S_I_ (r = −0.315, FDR =2.84E-02) in AA subjects. We found enrichment of inflammatory response pathway genes (p-value = 6.90E-09) among genes that negatively correlated with S_I_ only in CA subjects. Interestingly, a recent study in an animal model suggested that local pro-inflammatory response in adipose tissue is adaptive, required for proper adipose tissue remodeling and expansion to enable safe storage of excess nutrient and metabolic homeostasis [41].

Many adipose co-expression modules were enriched for genes differentially expressed between the two ethnicities or had differential connectivity (MDC) among members of the network module. Notably, the “bisque” module showed strong differential connectivity (MDC = 11.16) and was highly enriched for transcripts negatively correlated with S_I_ only in CAs. Driver genes of this sub-network (*OAS2, ISG15, STAT1,* and *ADAR*) are involved in immune response to viral infections, and expression of these genes is inducible by interferon. A recent personal-omics analysis showed transcriptional modulation of genes in pathways involved in glucose regulation of insulin secretion occurred shortly after viral infection, and was predicted as involved in elevation of glucose and HbA1c in a Caucasian subject [42]. A recent in vitro analysis showed significant ADAR genotype (and haplotype)-dependent modulation of IFNγ-producing cells (or IFNγ-Elispot responses upon viral infection) in CAs but not in AAs [43]. We showed that *ADAR* is one of the 7 CA-specific driver genes in this module. *IRF7*, another CA-specific driver of this module, was modulated by lipopolysaccharide, interferon, and virus-responsive *cis*-regulatory genetic variants in monocytes and dendritic cells [44,45]. *IRF7* modulates expression of several downstream genes in this pathway as a master *trans*-regulator. The “light yellow” module was enriched for transcripts correlated with S_I_ only in AAs. The top driver gene of this sub-network, *YWHAE* (14-3-3-epsilon protein), may play a role in the regulation of S_I_ by modulating the interaction between the insulin receptor and IRS1. However, the functions of many other genes in this sub-network are not clear.

Our study cohort included far more Caucasian subjects (N = 99) than African American subjects (N = 37). Unequal racial distribution in our cohort was influenced by the population demographics of Arkansas (recruitment site) and volunteer participation bias. However, to our knowledge, this is the largest single cohort of adipose tissue gene expression related to glucose homeostasis that includes phenotypically well-characterized subjects of both races. While sample size is an important factor for all analyses (e.g. differential expression, gene-trait correlation and network construction), the integrative network analysis was developed in this study to minimize the impact of a relatively small sample size, to obtain a more comprehensive understanding of differentially expressed genes and the network context under which individual genes operate. The highly significant overlap between the results from the two ethnic groups studied here and an independent but much larger study (the deCODE study) in Caucasian subjects suggests that the integrative network analysis approach was appropriate in balancing for the impact of the small sample size.

## Conclusions

By comparing co-expression networks in adipose tissue of metabolically well-characterized CA and AA subjects, we found derangements in transcriptional networks of genes involved in mitochondrial energy metabolism pathways that lead to insulin resistance in both ethnicities. We also found evidence that other molecular mechanisms involved in modulating glucose homeostasis are restricted predominantly to subjects from one ethnic origin. Distinct genetic (and epigenetic) regulatory architecture, including differences in the frequency of expression regulatory alleles among populations, may determine the structure of co-expression networks observed in adipose tissues of these subjects [46,47]. Further studies will be required to identify how genetic and epigenetic factors determine the structure of co-expression networks in adipose tissue that modulate glucose homeostasis and related physiological traits.

## Additional files

Additional file 1: Table S1. Genes showing differential expression in adipose tissue of African Americans compared to Caucasians. **Table S2.** Enrichment of functional category and biological pathway among genes showing differential expression in adipose tissue of African Americans compared to Caucasians. **Table S3.** Enrichment of functional category and biological pathway among genes correlated with insulin sensitivity (S_I_) in adipose tissue. **Table S4.** Weighted gene co-expression network analysis (WGCNA) identified 93 co-expression networks (modules) in adipose tissue of Caucasian subjects. **Table S5.** Correlation of module eigengenes with gluco- and cardio- metabolic phenotypes in Caucasian subjects. **Table S6.** Co-expression modules in adipose tissue of Caucasians are enriched for genes in functional categories and biological pathways. **Table S7.** Weighted gene co-expression network analysis (WGCNA) identified 173 co-expression networks (modules) in adipose tissue of African American subjects. **Table S8.** Correlation of module eigengenes with gluco- and cardio- metabolic phenotypes in African American subjects. **Table S9.** Co-expression modules in adipose tissue of African Americans are enriched for genes in functional categories and biological pathways. **Table S10.** A) Adipose tissue derived transcript modules in CA are enriched for genes differentially expressed in African Americans. B) Adipose tissue derived transcript modules in AA are enriched for genes differentially expressed in African Americans. **Table S11.** Co-expression modules in adipose tissue of Caucasians show differential connectivity. **Table S12.** Co-expression modules in adipose tissue of African Americans show differential connectivity. Additional file 2: Figure S1. Scale free topology plot of weighted adipose tissue co-expression network in (A) CA subjects (N = 99) and (B) AA subjects (N = 37) constructed with the power adjacency function power (s, beta = 5). Network satisfies a scale free topology approximately (black linear regression line R^2^ = 0.81. But a better fit is provided by an exponentially truncated power law (red line, R^2^ = 0.99).
