# Supplementary figures and images for: Integrative network analysis reveals different pathophysiological mechanisms of insulin resistance among Caucasians and African Americans
Source: BMC Med Genomics. 2015 Feb 7;8:4. doi: 10.1186/s12920-015-0078-0 (PMC4351975; doi:10.1186/s12920-015-0078-0)

## Slide 1
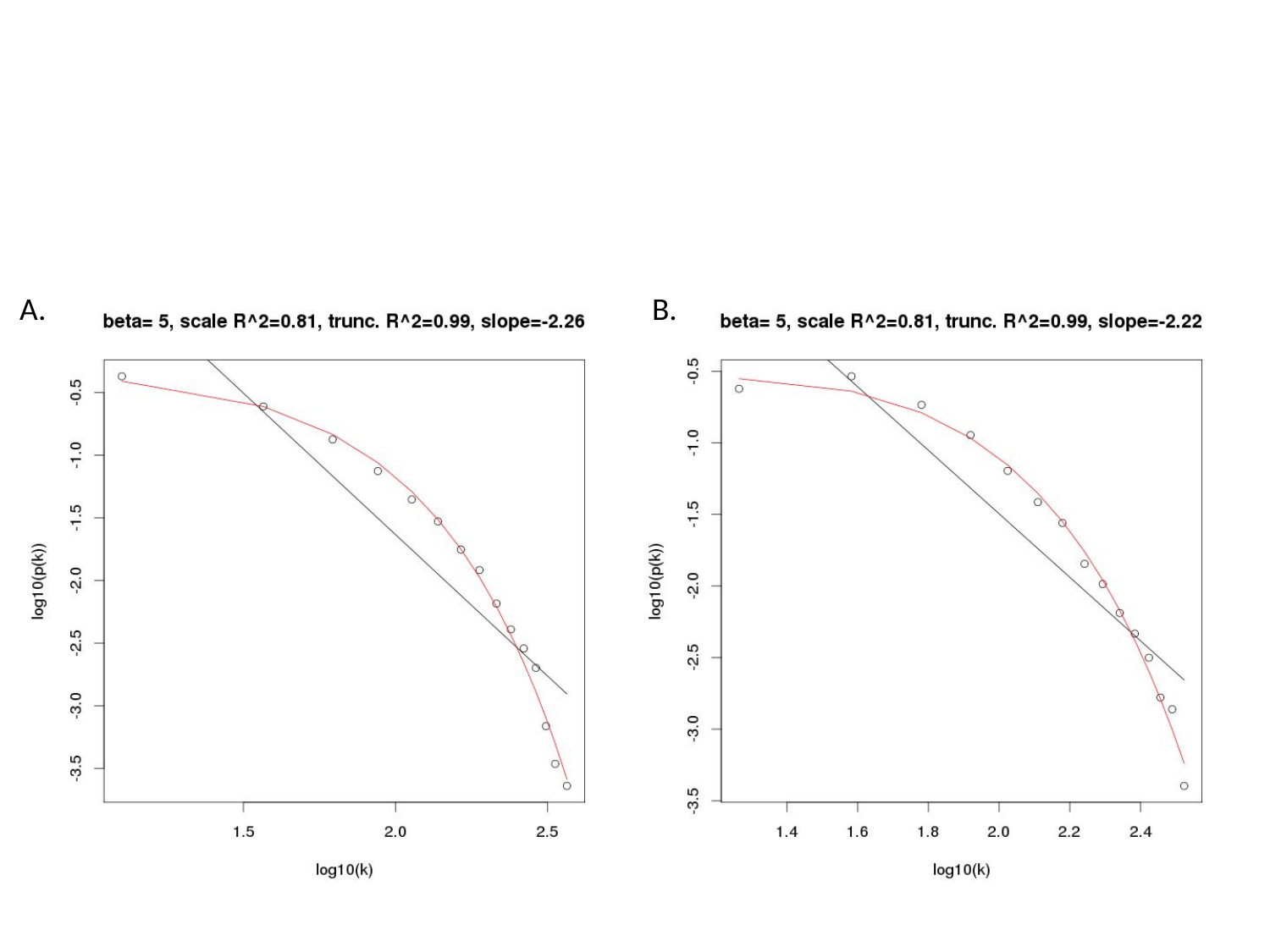

A.
B.

Supplement: Additional file 2: Figure S1. — Scale free topology plot of weighted adipose tissue co-expression network in (A) CA subjects (N = 99) and (B) AA subjects (N = 37) constructed with the power adjacency function power (s, beta = 5). Network satisfies a scale free topology approximately (black linear regression line R2 = 0.81. But a better fit is provided by an exponentially truncated power law (red line, R2 = 0.99). [file 12920_2015_78_MOESM2_ESM.pptx]
